# A randomised clinical trial to assess the adjuvant potential of methotrexate to corticosteroids in mucosal or limited mucocutaneous pemphigus vulgaris

**DOI:** 10.1038/s41598-022-11387-2

**Published:** 2022-05-09

**Authors:** Khimit Jain, Vishal Thakur, Sanjeev Handa, Neha Thakur, Shilpa, Naresh Sachdeva, Rahul Mahajan, Dipankar De

**Affiliations:** 1 Department of Dermatology, Venereology, and Leprology, Postgraduate Institute of Medical Education and Research, Sector 12, Chandigarh, 160012 India; 2 Department of Endocrinology (Immunology Division), Postgraduate Institute of Medical Education and Research, Chandigarh, 160012 India

**Keywords:** Diseases, Immunological disorders, Autoimmune diseases

## Abstract

Glucocorticoids are the mainstay of treatment for pemphigus vulgaris (PV). However, the requirement of high doses for long durations often leads to serious adverse events. Methotrexate as an adjuvant has shown potential in retrospective studies but randomized trials are lacking. The objective of the study was to assess the adjuvant potential of methotrexate in mucosal/limited mucocutaneous PV. In this randomised prospective study, 44 patients with mucosal/limited mucocutaneous PV were randomised (1:1) to receive either prednisolone 1 mg/kg/day (later fixed at a maximum dose of 60 mg/day) alone or with methotrexate 0.3 mg/kg/week for 9-months study period. Prednisolone dose was tapered once there was an 80% reduction in Pemphigus Disease Activity Index. Outcome measures were total cumulative dose of prednisolone, the proportion of patients achieving disease control, time taken for disease control and remission on minimal treatment, and adverse effects. No significant difference in the total cumulative dose of prednisolone among the groups was observed (p = 0.68). Disease control was achieved in 95.5% and 86.4% of patients in the prednisolone alone group, and prednisolone and methotrexate group respectively (p = 0.61). No statistically significant difference was observed among the groups with respect to the proportion of patients achieving remission, time taken for disease control and remission, and the number of adverse events. Our study showed no additional benefit of methotrexate to prednisolone in the treatment of mucosal/limited mucocutaneous PV.

**Trial registration:** CTRI/2018/07/015002; Registered on 23/07/2018]; Trial Registered Retrospectively. http://ctri.nic.in/Clinicaltrials/pdf_generate.php?trialid=24964&EncHid=&modid=&compid=%27,%2724964det%27.

## Introduction

Pemphigus vulgaris (PV) is a chronic autoimmune blistering disorder with considerable morbidity and mortality. Glucocorticoids remain the mainstay for the treatment of PV. However, the high dosages of corticosteroids required and prolonged use are associated with frequent complications and morbidity^1^. The primary goal for the treatment of PV should be to induce and maintain remission of disease with the lowest possible cumulative steroid dose^2^. Therefore, adjuvants to prednisolone such as azathioprine, mycophenolate mofetil (MMF), rituximab, cyclophosphamide, or other immunosuppressants/immunomodulators are used depending on availability, prescription practice, and disease severity.

Methotrexate is one of the first immunosuppressive agents other than corticosteroids to be used in PV. Methotrexate is used in lower doses in patients with autoimmune and chronic inflammatory diseases like psoriasis and rheumatoid arthritis. The low dose used provides significant clinical relief and is associated with significantly reduced toxicity^3,4^. Observations on methotrexate indicate that the anti-inflammatory effects may be of greater magnitude than the immunosuppressive effects^5^. This valuable aspect of methotrexate may be one reason for the lower incidence of infections in patients on methotrexate compared with those on cyclophosphamide, azathioprine, MMF, or ciclosporin^5^. Thus, low-dose methotrexate can be a preferred adjuvant for PV patients with limited financial resources and/ or where other drugs are contraindicated. Considering the lack of a randomized controlled trial on methotrexate in PV, we assessed the adjuvant potential of methotrexate on the required cumulative dosage of steroids in mucosal or limited mucocutaneous PV over a defined period.

## Methods

This was a single-center, randomized prospective study conducted at a tertiary care center in India from January 2018 to March 2020. The study was approved by the Institutional Ethics Committee (Intramural), Post Graduate Institute of Medical Education and Research, Chandigarh, India and registered on the Clinical Trials Registry of India (CTRI/2018/07/0150002). A total of 44 patients satisfying inclusion and exclusion criteria were included in the study. After obtaining written informed consent and baseline investigations, patients were randomized (1:1) using a computer-generated randomization sequence to receive either prednisolone alone or prednisolone with methotrexate 0.3 mg/kg/week (maximum dose of 25 mg/week). Patients were screened for eligibility by Jain and recruited by Handa, Mahajan, and De. Allocation to either group was done by De following which treatment was prescribed such that patients were not blinded to the treatment received. At follow-up, the assessment was done by Jain who was blinded to randomization and treatment allocation.

### Sample size estimation

The rationale behind the addition of methotrexate to prednisolone as an adjuvant is to reduce the total cumulative dose of prednisolone, thereby reducing its dose-dependent side effects. We assumed a difference of 25% in the total cumulative dose of prednisolone between the 2 groups. The assumed standard deviation for the drug was taken as 40% with the power of study at 80% and a significance level of 5%. Considering the dropout rate of 10%, the total sample size was calculated to be 44 patients (22 in each arm).

### Inclusion and exclusion criteria

Patients of either gender aged > 18 years having PV with oral mucosal lesions with no or limited cutaneous involvement (i.e. < 5% body surface area involvement) and willing for biweekly follow up for the first month and monthly follow up visits for at least 8 months thereafter were included in the study. Exclusion criteria were pregnant and lactating females, elderly patients aged 70 years or older, any absolute/relative contraindication to methotrexate and/or prednisolone, and hypersensitivity to methotrexate.

### Outcome measures

The primary outcome measure was to compare the total cumulative dose of prednisolone required over 9 months study period between the groups. Secondary outcome measures were the proportion of patients achieving disease control, time taken for disease control, overall remission rate, time to remission with minimal treatment, and side effects of treatment. Disease control for this study was arbitrarily defined as 80% reduction in Pemphigus Disease Activity Index (PDAI)^6^ and no new lesions in the previous four weeks. The ‘complete remission on minimal therapy’ was defined as the absence of new or established lesions while the patient is receiving minimal therapy (≤ 10 mg/day of prednisolone). The definition of ‘minimal therapy’ was based solely on the prednisolone dose as the dose of methotrexate was maintained throughout the study period in the group receiving it.

### Patient assessment

Forty-four consenting patients were consecutively enrolled in the study. Diagnosis of PV was based on clinical findings, Tzanck smear, histopathology, and direct immunofluorescence. Detailed history regarding duration, distribution, the severity of illness were recorded. A baseline disease severity (assessed by PDAI), body weight and blood pressure were documented. Baseline investigations including complete blood count, renal function test, liver function test, urine analysis, chest X-ray, viral markers (hepatitis B/C serologies and HIV), random blood sugar, and urine pregnancy test (if applicable) were done.

**Figure 1:**
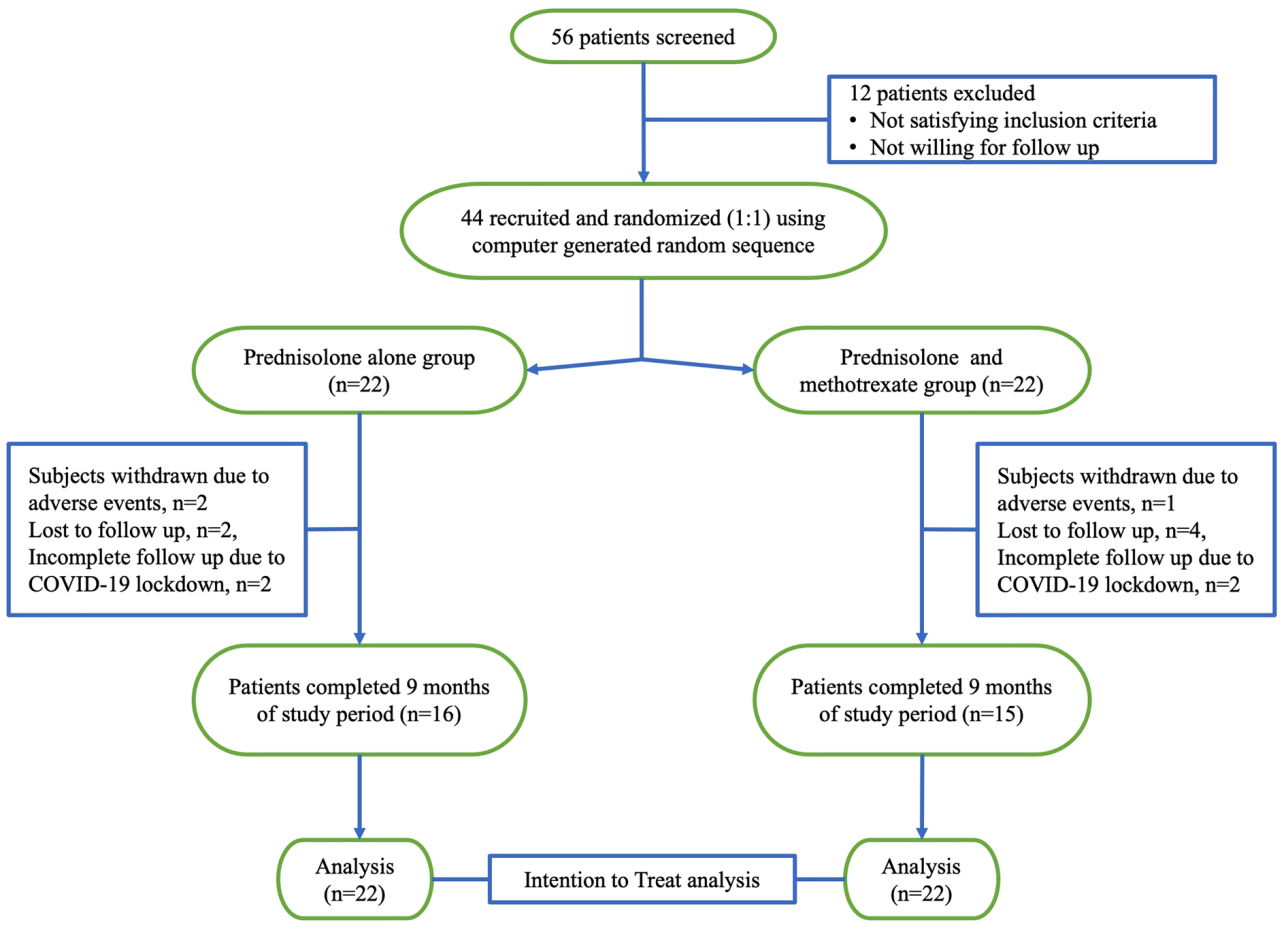
CONSORT diagram of study participants with inclusion and exclusion of patients and allocation of patients into groups.

In the prednisolone alone group, prednisolone was prescribed at a dose of 1 mg/kg/day (rounded off to nearest multiple of 5, fixed later at a maximum dosage of 60 mg/day). Prednisolone dose was tapered once there was an 80% reduction in PDAI and no new lesions appeared in the previous 4-weeks. The dose was reduced by 10 mg every 2-weeks until 20 mg/day and 5 mg every 2-weeks thereafter. After 10 mg/day, the dose was reduced by 2.5 mg on alternate weeks. In case of disease flare-up defined as ≥ 3 point increase in PDAI score in a patient who has achieved disease control; the prevailing dose of prednisolone was increased by 5–10 mg and was subsequently reduced as per the slab once disease control is achieved. In prednisolone and methotrexate group, the schedule of prednisolone dosing was similar to the prednisolone alone group and methotrexate was given at a dose of 0.3 mg/kg/week (rounded off to nearest multiple of 2.5, up to a maximum dosage of 25 mg/week) along with 2.5 mg of folic acid a day before and after methotrexate. The dose of methotrexate was kept constant until the completion of the 9-months study duration. All subjects were reviewed biweekly for the first month and monthly for the next 8 months. A full clinical examination was performed during each visit including general physical examination, assessment of PDAI, and assessment of treatment-related adverse effects. Follow-up investigations included standard methotrexate monitoring protocol and random blood sugar every month. In case of side effects requiring treatment discontinuation or in case of disease progression, the patient was withdrawn from the study and managed appropriately. A flowchart depicting study participants with inclusion and exclusion of patients is given in Fig. 1.

### Statistical analyses

Data were analysed using SPSS software version 25.0 (IBM Corp., Armonk, NY, USA). The normalcy of variables was checked by Kolmogorov–Smirnov Test. For normally distributed data, means were compared using Student’s *t* test for two groups. For skewed/ordinal data, Mann–Whitney test was applied for two groups. Qualitative or categorical variables are represented as frequencies and proportions. Proportions were compared using Chi-square or Fisher’s exact test, whichever was applicable. All statistical tests were two-sided and were performed at a significance level of α < 0.05.

### Institutional ethical committee approval

This study was performed in line with the principles of the Declaration of Helsinki. Approval was granted by the Institutional Ethics Committee (Attached as supplementary file).

### Consent to participate

Informed consent was obtained from all individual participants included in the study.

## Results

Of 44 patients, 3 patients were withdrawn from the study. Two patients in the prednisolone alone group were withdrawn- one was withdrawn at 3 months due to disease progression and the other at 8 months due to tuberculosis. One patient in the prednisolone and methotrexate group was withdrawn at 4 months due to severe transaminitis. Data were analysed using the intention-to-treat method. Baseline clinicodemographic data of patients in both groups are summarized in Table 1 and were comparable. All patients had mucocutaneous PV with mucosal onset of disease in 34 (77.3%) patients and buccal mucosa was the commonest site of onset among oral mucosa (72.7%). Mean body weight was comparable among the groups—59.63 ± 11.1 kg in the prednisolone alone group vs 59 ± 10.1 kg in the prednisolone and methotrexate group (p = 0.84). Baseline PDAI in prednisolone alone group and prednisolone and methotrexate group were 29 ± 16.4 and 23.6 ± 10.1, respectively (p = 0.21). Previous treatments included corticosteroids, azathioprine, rituximab, intravenous immunoglobulin and oral antibiotics. The patients included in the study had received rituximab at least 6 months or IVIG 3 months before randomization. Azathioprine was stopped at the screening visit. Table 2 shows the comparison of the outcomes between the two groups. There was no significant difference in the total cumulative dose of prednisolone among the groups at the end of the study period (6725.8 ± 3103.05 mg in prednisolone alone group vs 6308.59 ± 3483.66 mg in prednisolone and methotrexate group; p = 0.68). Similarly, cumulative prednisolone dose/kg body weight was also comparable between the groups (p = 0.78). Disease control was achieved in 95.5% and 86.4% of patients in the prednisolone alone group, and prednisolone and methotrexate group respectively (p = 0.61). The mean time for disease control was 10.62 ± 5.6 and 11.42 ± 6.35 weeks in prednisolone alone group, and prednisolone and methotrexate group respectively (p = 0.67). Remission was achieved in 54.5% and 50% in the prednisolone alone group, and prednisolone and methotrexate group respectively (p = 1.0). Mean time taken for remission was 18.83 ± 7.8 and 20.36 ± 11.39 weeks in prednisolone alone group, and prednisolone and methotrexate group respectively (p = 0.71). No statistically significant difference was observed among the groups with respect to the time taken for disease control and time taken for remission. Complete remission on minimal therapy was achieved in only 3 (13.6%) patients in prednisolone alone group and 2 (9.1%) in prednisolone and methotrexate group (p = 1.0) and time taken for the same was 25.67 ± 2.08 weeks in prednisolone alone group and 30 ± 8.49 weeks in prednisolone and methotrexate group (p = 0.43). Cumulative prednisolone dose until complete remission on minimal therapy was less in the prednisolone and methotrexate group (3867.5 ± 668.22 mg) as compared to the prednisolone alone group (5125 ± 1192.42 mg). However, the difference was not statistically significant (p = 0.28).

**Table 1:** Comparison of baseline clinicodemographic characteristics of the groups.

Characteristic	Prednisolone alone group; n = 22	Prednisolone + methotrexate group; n = 22	p-value
Age (years); mean ± SD	40.86 ± 15.69	43 ± 11.61	0.61
Sex (M:F)	13:9	8:14	0.23
Bodyweight (kg); mean ± SD	59.63 ± 11.1	59 ± 10.1	0.84
Total duration of disease (months); mean ± SD	14.4 ± 21.37	17.98 ± 20.44	0.62
Total duration of mucosal disease (months); mean ± SD	13.46 ± 20.44	14.7 ± 25.2	0.86
Baseline PDAI, mean ± SD	29 ± 16.4	23.6 ± 10.1	0.21
**Site of onset (%)**			
Mucosa	19 (86.4)	15 (68.2)	0.15
Skin	3 (13.6)	7 (31.8)	
Other mucosal involvement (%)	9/22 (40.9)	10/22 (45.4)	1.0
History of smoking (%)	2/22 (9.1)	1/22 (4.5)	1.0
History of alcohol consumption (%)	1/22 (4.5)	1/22 (4.5)	1.0
**Previous treatment (%)**			
Corticosteroids	18 (81.8)	19 (86.4)	0.23
Azathioprine	3 (13.6)	0	
Rituximab	1 (4.5)	2 (9.1)	
Oral antibiotics	1 (4.5)	1 (4.5)	
IVIG	0	1 (4.5)

**Table 2:** Comparison of outcome measures between the groups.

Characteristic	Prednisolone alone group; n = 22	Prednisolone + Methotrexate group; n = 22	p-value
Total cumulative prednisolone dose (mg); mean ± SD	6725.8 ± 3103.05	6308.59 ± 3483.66	0.68
Cumulative prednisolone dose (mg)/kg body weight; mean ± SD	113.14 ± 44.59	106.97 ± 52.93	0.68
Number of patients achieving disease control (%)	21 (95.5)	19 (86.4)	0.61
Time taken for disease control (weeks); mean ± SD	10.62 ± 5.6	11.42 ± 6.35	0.67
Cumulative prednisolone dose until disease control (mg); mean ± SD	4287.14 ± 2600.69	4339.74 ± 2763.74	0.95
Number of patients achieving remission (%)	12 (54.5)	11 (50)	1.0
Time taken for remission (weeks); mean ± SD	18.83 ± 7.8	20.36 ± 11.39	0.71
Cumulative prednisolone dose until remission (mg); mean ± SD	6228.17 ± 3892.14	6128.18 ± 3650.01	0.95
Number of patients achieving complete remission on minimal therapy (%)	3 (13.6)	2 (9.1)	1.0
Time taken for complete remission on minimal therapy (weeks); mean ± SD	25.67 ± 2.08	30 ± 8.49	0.43
Cumulative prednisolone dose until complete remission on minimal therapy (mg); mean ± SD	5125 ± 1192.42	3867.5 ± 668.22	0.28
Number of patients having disease flare (%)	5 (22.7)	4 (18.2)	1.0
Number of adverse events; mean ± SD	3.68 ± 2.87	4.24 ± 2.96	0.54

Disease flare was seen in 5 (22.7%) and 4 (18.2%) patients in the prednisolone alone group and prednisolone and methotrexate group, respectively (p = 1.0). Two patients in each group had 2 episodes of disease flare while disease flare occurred once in 3 patients in the prednisolone alone group and 2 patients in the prednisolone and methotrexate group.

The mean number of adverse events that occurred in both groups was comparable (3.68 ± 2.87 in the prednisolone alone group vs 4.24 ± 2.96 in the prednisolone and methotrexate group respectively; p = 0.54). Common adverse events in both groups included facial mooning, muscle cramps, acneiform eruptions, sleeplessness, and weight gain.

## Discussion

Only a few retrospective studies have assessed the use of methotrexate in PV and no randomised prospective study has been done to assess the adjuvant potential of methotrexate in PV. Given the lack of data, the British Association of Dermatologists guideline recommends the use of methotrexate in PV only when other steroid-sparing agents have failed or cannot be used^7^. However, the European Academy of Dermatology and Venereology (EADV) guideline does not recommend methotrexate in the treatment of PV^2^.

In our study, patients who received the combination of prednisolone and methotrexate, did not require a lower cumulative dose of prednisolone to control their disease, and did not achieve disease control and remission more often and earlier than those treated with corticosteroids only; suggesting no benefit of methotrexate as an adjuvant to prednisolone in the treatment of PV. This was in contrast to earlier retrospective studies which showed up to or > 50% decrease in total cumulative dose of prednisolone when used in combination with methotrexate^8,9^. A retrospective chart review of pemphigus patients treated with methotrexate reported complete cessation of steroids in 70% of patients after a mean duration of 18 months^10^. However, steroids were discontinued in 6 of 9 patients within 6 months of therapy without any flare in another retrospective study^11^. A systematic review in 2009 of 136 PV patients treated with methotrexate alone or in combination with prednisolone and/or other immunosuppressants found that 82% showed clinical improvement with methotrexate^5^. A retrospective chart review of 30 patients with PV treated with methotrexate 15 mg/week showed clinical improvement in 84% of patients and reduced prednisolone dose in 77% of patients^9^. In another retrospective analysis, 14 out of 41 patients achieved remission off treatment^12^. The authors concluded that methotrexate is ineffective in severe PV. In patients with severe or moderately severe PV in whom the disease had been brought under control by high doses of prednisolone, methotrexate has only a minimal and often delayed beneficial effect on oral lesions, whereas the cutaneous lesions usually respond fairly well with the reduction in the required maintenance doses of prednisolone^13^. Another study showed significant improvement in 8 of 9 pemphigus patients treated with methotrexate and steroids but had significant adverse effects in 6 patients^14^. All these studies were retrospective in nature, associated with their inherent biases and limited available data. These limitations were also highlighted by Gürcan et al. in their review of 136 patients treated with methotrexate alone or in combination with prednisolone^5^. Moreover, standard definitions for disease severity or outcomes were not introduced when these studies were undertaken and outcome parameters were also different.

Given the lack of randomised studies assessing methotrexate as a steroid-sparing agent, it is difficult to conduct a direct comparison with published studies whereas comparison with other agents seems feasible. Azathioprine is the most commonly used steroid-sparing immunosuppressant in PV. In a study, azathioprine in combination with prednisolone was found more effective than prednisolone alone in terms of reduction in disease activity. However, no difference was found in the total prednisolone dose received in both groups in the first 9 months of the study^15^. Similarly, in another randomized controlled open-label study, azathioprine along with prednisolone was found more effective than prednisolone alone, MMF with prednisolone, and cyclophosphamide pulse in combination with prednisolone in terms of steroid-sparing effect. However, no difference was observed in rates of disease remission among the groups^16^.

Another immunosuppressive agent, MMF, when used in combination with prednisolone was not found to have any steroid-sparing effect and adverse events were more frequent with the combination^17^. Findings similar to our study have been observed in a randomised prospective study comparing prednisolone alone with prednisolone and MMF combination. Complete remission on therapy was achieved in 52.17% and 54.17% patients in prednisolone alone and prednisolone and MMF group, respectively, at the end of 1-year^17^. Time taken for remission was similar to our study^17^. This study also demonstrated that MMF was not an effective adjuvant and no significant reduction in the total dose of prednisolone was observed. The rates of remission with MMF have also been similar to that observed with prednisolone alone in different studies and remission was not achieved early with MMF^16–19^.

The adverse events observed in our study were comparable among the groups and mostly could be ascribed to prednisolone. This may be due to the lower doses of methotrexate used in our study. Previous studies with methotrexate have shown significant adverse effects. Studies with other steroid-sparing agents have documented significant adverse effects with these agents as compared to prednisolone alone^8,15,16,18,20^.

The short study period and follow-up are the limitations of this study. However, as the ultimate aim of treatment in PV is the maintenance of disease remission with the lowest possible dose of prednisolone, and assessment of total cumulative dose of prednisolone in a defined but reasonably long duration was the primary aim of the study; the results presented here are of importance.

## Conclusion

The findings of our study suggest that methotrexate offers no advantage when added to prednisolone in the treatment of PV. The use of methotrexate did not have any significant additional adverse effects over prednisolone.

## Data Availability

The datasets generated and/or analyzed during the current study are available from the corresponding author on reasonable request.
